# Virulence genes are a signature of the microbiome in the colorectal tumor microenvironment

**DOI:** 10.1186/s13073-015-0177-8

**Published:** 2015-06-24

**Authors:** Michael B. Burns, Joshua Lynch, Timothy K. Starr, Dan Knights, Ran Blekhman

**Affiliations:** Department of Genetics, Cell Biology, and Development, University of Minnesota, Minneapolis, MN USA; Department of Ecology, Evolution, and Behavior, University of Minnesota, Minneapolis, MN USA; Masonic Cancer Center, University of Minnesota, Minneapolis, MN USA; Department of Obstetrics, Gynecology and Women’s Health, University of Minnesota, Minneapolis, MN USA; Department of Computer Science and Engineering, University of Minnesota, Minneapolis, MN USA; Biotechnology Institute, University of Minnesota, Minneapolis, MN USA

## Abstract

**Background:**

The human gut microbiome is associated with the development of colon cancer, and recent studies have found changes in the microbiome in cancer patients compared to healthy controls. Studying the microbial communities in the tumor microenvironment may shed light on the role of host–bacteria interactions in colorectal cancer. Here, we highlight the major shifts in the colorectal tumor microbiome relative to that of matched normal colon tissue from the same individual, allowing us to survey the microbial communities in the tumor microenvironment and providing intrinsic control for environmental and host genetic effects on the microbiome.

**Methods:**

We sequenced the microbiome in 44 primary tumor and 44 patient-matched normal colon tissue samples to determine differentially abundant microbial taxa These data were also used to functionally characterize the microbiome of the cancer and normal sample pairs and identify functional pathways enriched in the tumor-associated microbiota.

**Results:**

We find that tumors harbor distinct microbial communities compared to nearby healthy tissue. Our results show increased microbial diversity in the tumor microenvironment, with changes in the abundances of commensal and pathogenic bacterial taxa, including *Fusobacterium* and *Providencia*. While *Fusobacterium* has previously been implicated in colorectal cancer, *Providencia* is a novel tumor-associated agent which has not been identified in previous studies. Additionally, we identified a clear, significant enrichment of predicted virulence-associated genes in the colorectal cancer microenvironment, likely dependent upon the genomes of *Fusobacterium* and *Providencia*.

**Conclusions:**

This work identifies bacterial taxa significantly correlated with colorectal cancer, including a novel finding of an elevated abundance of *Providencia* in the tumor microenvironment. We also describe the predicted metabolic pathways and enzymes differentially present in the tumor-associated microbiome, and show an enrichment of virulence-associated bacterial genes in the tumor microenvironment. This predicted virulence enrichment supports the hypothesis that the microbiome plays an active role in colorectal cancer development and/or progression. Our results provide a starting point for future prognostic and therapeutic research with the potential to improve patient outcomes.

**Electronic supplementary material:**

The online version of this article (doi:10.1186/s13073-015-0177-8) contains supplementary material, which is available to authorized users.

## Background

Colorectal cancer (CRC) is the second most commonly diagnosed cancer in females and the third in males worldwide [1]. The microbial communities present in the intestinal tract have known associations with colon health, though until recently researchers were limited to the study of microbes that were amenable to in vitro culturing. As a result of recent advances in culture-independent measurements of microbial communities, we know that the human gut is host to roughly a thousand different bacterial species [2]. Alterations of this bacterial community are correlated with host health, including diseases ranging from diabetes and obesity to Crohn’s disease and arteriosclerosis [3]. The composition of the gut microbiome also has a known association with CRC, although the direction of causality remains unclear [4–10]. A recent report demonstrated that analysis of the microbiome can be used as a pre-screening test for CRC that dramatically outperforms the current standard methods [11]. These analyses have identified significant shifts in the relative abundances of specific bacterial taxa in CRC cancer patients’ colon mucosa and stool microbiomes. For instance, bacteria in the genus *Fusobacterium* are enriched in some CRC patients’ microbiomes [7, 8, 10, 12]. *Fusobacterium* are thought to elicit a pro-inflammatory microenvironment around the tumor, driving tumor formation and/or progression [7]. More specifically, a recent study has demonstrated that the FadA protein, a virulence factor expressed by *Fusobacterium nucleatum*, can signal epithelial cells via E-cadherin, a cell-surface molecule important for CRC metastasis as well as a component of the WNT/β-catenin signaling pathway, the most commonly mutated pathway in CRC [13, 14]. Other cancer-associated bacterial taxa have been identified, including *Escherichia coli* strain NC101 and *Bacteroides fragilis*, each with a proposed mechanism of interaction with colon cancer [15]. The species *Akkermansia mucinphila*, a bacterium that has known associations with obesity, has also been implicated as a cancer-associated agent, with its mucin-degrading activity as a proposed mechanism to drive inflammation contributing to cancer genesis and/or progression [15].

In addition to defining the set of bacteria significantly associated with CRC, several groups have used measurements of microbiome diversity to compare cancer patients with normal subjects. There are distinct differences in these results that depend on the sources of the samples used to assess the microbiome (e.g., stool samples versus mucosal or tissue samples, longitudinal versus cross-sectional sampling). For instance, in a study that used stool samples to compare CRC patients with normal controls, the researchers showed decreased alpha-diversity among the microbial communities found in the CRC patients’ stools compared with the control [16]. Another, recent study, also focusing on fecal samples, was unable to detect differences in microbial community diversity or richness between normal and cancer-associated microbiomes [17]. However, in a study that used tissue samples from patients with colon adenomas and compared them with patient-matched normal tissues, the alpha-diversity present at the site of the lesion was actually increased [18]. This finding was repeated by Mira-Pascual et al. [19], who performed side-by-side analyses of tissue and stool samples. They found that stool samples in general had roughly twice the microbial diversity when compared with tissue-associated microbiomes, with stools from cancer patients showing lower diversity than those from normal controls. When comparing the microbial diversity between tissue samples, however, the tumor microbiome was more diverse than the normal microbiome. It is likely that this is a function of the stool samples harboring microbes from the entire colonic environment, including species that are not directly related to the tumor microenvironment, adding noise to the taxonomic results acquired from assessment of stool samples relative to direct measurements of tissues. These findings suggest that in order to detect differences specific to the cancer-associated microbiome, samples taken directly from the tumor microenvironment are preferable, at least at the initial characterization phase, to bulk stool samples, which are not likely to have the discriminatory power required to measure small, yet significant, effects [19].

The use of traditional case-control studies of the colon cancer microbiome makes it difficult to control for all of the external effects on the microbiome. For example, the composition of gut microbial communities is strongly affected by diet [20]. Host genetic variation is also expected to control variation in the gut microbiome [21], through differences in host immune response among other genetic mechanisms [22]. The large effects these factors have on microbiome composition are likely to confound traditional case-control studies. By using tumor and normal tissue samples taken from the same individual, our study controls for these variables internally, providing a more accurate view of the tumor-associated shifts in the microbiome. Several previous studies have utilized this strategy to assess changes in the cancer-associated microbiome in a variety of populations [7, 9, 10, 12, 19, 23, 24]. However, most used far fewer samples than would be necessitated by using unmatched tissue or stool samples. Another caveat with these analyses, even with the use of patient-matched samples, is that the normal tissue itself may possibly be affected by the changes elicited in the organ by the tumor. There has been a report indicating that, in some cases, biofilms may form in otherwise normal areas of the colon of patients who also have tumors [24]. In the event that this is the case, the overall differences seen between tumor and normal matched tissue microbiomes might be diminished as the tumor is producing a “field effect” that influences surrounding tissue.

In addition to measuring bacterial taxa levels in colon cancer, it is also important to take into account associated factors, such as host genetics and gene expression, as well as the microenvironmental metabolome. Independent research groups have attempted to uncover pertinent alterations in these factors and how they correlate with cancer state [25–27]. Of note, analysis of the CRC-associated metabolome highlighted differences in the biochemical composition of cancer patients’ stools. CRC patients were found to have higher levels of some amino acids and alterations in the levels of some short chain fatty acids (SCFAs) in their stools when compared with controls [25]. Butyrate, a SCFA with known anti-cancer properties, was depleted in CRC patient stool samples, as were several genera of butyrate-producing bacteria [28]. Our work continues this effort by expanding the analysis of the CRC-associated microbiome to include virtual metagenomic profiling of the enzymes and pathways present in the CRC microbiome, with specific attention paid to assessing the presence of known virulence-associated genes [29]. Previous work has been performed attempting to identify transcripts from 22 different, potentially cancer-related bacterial toxins in metatranscriptomic data, though with limited success [30]. As this study focuses on a comparison of fully developed tumors and compares them with patient-matched normal tissue, it is important to highlight that this work is not an assessment of the initiation of cancer, but rather a characterization of cancer after it has transformed into a malignancy.

## Methods

### Tissue samples and DNA isolation

We used 88 tissue samples from 44 individuals, with one tumor and one normal sample from each individual. These de-identified samples were obtained from the University of Minnesota Biological Materials Procurement Network (Bionet), a facility that archives research samples from patients who have provided written, informed consent. All research conformed to the Helsinki Declaration and was approved by the University of Minnesota Institutional Review Board, protocol 1310E44403; see Additional file 1 for detailed information on the samples used in this study. Tissue pairs were resected concurrently, rinsed with sterile water, flash frozen in liquid nitrogen, and characterized by staff pathologists. The criteria for selection were limited to the availability of patient-matched normal and tumor tissue specimens. The specific site of the tumor within the intestinal tract was recorded and can be found in Additional file 1. Total DNA was isolated from the flash-frozen tissue samples and their associated microbiomes by adapting an established nucleic acid extraction protocol [31]. Briefly, approximately 100 mg of flash-frozen tissue were physically disrupted by placing the tissue in 1 mL of Qiazol lysis solution and sonicating in a heated (65 °C) ultrasonic water bath for 1–2 h. The efficiency of this approach was verified by observing high abundances of Gram-negative bacteria across all samples, including those from the phylum Firmicutes. Additionally, sequences from the notoriously difficult to lyse bacterial genera *Mycobacterium* and/or *Bacillus* [32] were detected in the majority of samples, also indicating a rigorous and efficient lysis. DNA was purified from the lysate using the Qiagen All-prep kit (Qiagen Inc., Valencia, CA, USA).

### 16S rRNA sequencing

Briefly, DNA isolated from colon samples was quantified by quantitative PCR (qPCR), and the V5-V6 regions of the 16S rRNA gene were PCR amplified with the addition of barcodes for multiplexing. The forward and reverse primers were the V5F and V6R sets from Cai et al. [33]. The PCR conditions were as follows. Amplification was carried out in a 25 μL PCR reaction with 5 μL of template DNA with an initial denaturation step at 95 °C for 5 min followed by 30 cycles of denaturation (50 s at 94 °C), annealing (30 s at 40 °C), and elongation (30 s at 72 °C). Amplified samples were then diluted 1:100 in water for input into library tailing PCR. This PCR reaction was similar to initial amplification except the PCR conditions consisted of an initial denaturation at 95 °C for 5 min followed by 15 cycles of denaturation (50 s at 94 °C), annealing (30 s at 40 °C), and elongation (1 min at 72 °C). Quantification of PCR products was performed using the Quant-iT PicoGreen dsDNA Assay Kit (Life Technologies, Grand Island, NY, USA). A subset of the amplified libraries was spot-checked on a Bioanalyzer High-Sensitivity DNA Chip (Agilent Technologies, Santa Clara, CA, USA) to ascertain if the amplicons were the predicted size. These samples were each normalized to 2 nM and pooled. The total volume of the libraries was reduced using a SpeedVac and amplicons were size-selected at 420 bp ±20 % using the Caliper XT (Perkin Elmer, Waltham, MA, USA). The pooled libraries were cleaned with 1.8× AMPureXP beads (Beckman Coulter, Brea, CA, USA) and eluted with water. DNA concentration in the final pool was assayed with PicoGreen and normalized to 2 nM for input into Illumina MiSeq (v3 Kit) to produce 2 × 250 bp sequencing products. Clustering was performed at 10 pM with a 5 % spike of PhiX. A single lane on an Illumina MiSeq instrument was used to generate the 16S rRNA gene sequences. Raw sequencing data have been submitted to the NCBI Sequence Read Archive under project accession PRJNA284355.

### PCR and qPCR

Quantitative real-time PCR was performed to assess the abundance of the *FadA* gene present in a subset of normal and tumor tissue pairs. DNA from the ATCC control strain of *F. nucleatum* 25586 was used as a positive control. *FadA* abundances were normalized relative to pan-eubacteria abundance per sample. Primers FadA-F (5′-GAAGAAAGAGCACAAGCTGA-3′) and FadA-R (5′-GCTTGAAGTCTTTGAGCTCT-3′) were used to measure *FadA* [14], and primers for universal eubacteria 16S (5′-GGTGAATACGTTCCCGG-3′) and (5′-TACGGCTACCTTGTTACGACTT-3′) were used to determine the total eubacterial abundance per sample [34]. The analysis was performed using 10 ng of DNA in a 20 μL reaction containing FastStart Universal SYBR Green Master Mix (Rox; Roche Diagnostics, Indianapolis, IN, USA) on an Applied Biosystems 7300 Real Time PCR system. Reactions were performed in triplicate. *FadA* relative abundances were calculated as per the ΔCT method [35]. Relative fold differences were calculated by dividing the *FadA* abundance from the normal samples by that of the tumor sample.

*Fusobacterium* genus-specific PCR was performed on a subset of samples using previously characterized primers: forward (5′-GGATTTATTGGGCGTAAAGC-3′) and reverse (5′-GGCATTCCTACAAATATCTACGAA-3′) [34, 36]. The PCR was carried out using Accustart Taq polymerase (Quanta Biosciences, Gaithersburg, MD, USA) following the manufacturer’s protocol for 30 cycles with an annealing temperature of 55 °C. DNA from the ATCC control strain *F. nucleatum* 25586 was used as a positive control.

*Providencia* genus-specific PCR was performed using a previously published protocol and primer set: sp16s-F1 (5′-ACCGCATAATCTCTTAGG-3′) and Psp16s-R2 (5′-CTACACATGGAATTCTAC-3′), with the following modifications [37]. The PCR was carried out using Accustart Taq polymerase (Quanta Biosciences, Gaithersburg, MD, USA) following the manufacturer’s protocol for 30 cycles with an annealing temperature of 50 °C. The ATCC control strain *Providencia alcalifaciens* 9886 was used as a positive control. Amplicons were resolved in 2 % agarose TAE gel.

### Sequence analysis

The sequence data contained approximately 21.4 million reads passing quality filtering in total, inclusive of forward and reverse reads, with a mean value of 242,940 quality reads per sample. The forward and reverse read pairs were merged using the USEARCH v7 program ‘fastq_mergepairs’, allowing stagger, with no mismatches allowed [38]. Merged reads were quality trimmed, again using USEARCH, to truncate reads at any quality scores of 20 or less. Following merging and trimming, there were an average of 62,100 high quality reads per sample (median 48,817; range 6559–173,471). The fasta sequence headers were renamed using a custom script to conform to QIIME standards.

The merged and filtered reads were used to pick operational taxonomic units (OTUs) with QIIME v.1.7.0 using ‘pick_otus.py’, with the closed-reference usesearch_ref OTU picking protocol against the Greengenes database (August 2013 release) at 97 % similarity [39–41]. Reverse read matching was enabled, while reference-based chimera calling was disabled. Rarefaction was performed on the OTU table at 5000 reads prior to subsequent analyses.

The final OTU table was used to generate a phylogenetic tree by including only taxa with at least 0.1 % relative abundance in at least half of all samples. Starting with the full reference tree provided by the Greengenes database (August 2013 release, file 97_otus_unannotated.tree), a smaller tree file that contained only this limited set of taxa was generated using a custom pipeline (Sycamore from the Alm laboratory at MIT). The output of this pipeline was visualized with the Interactive Tree of Life [41, 42]. See Additional file 2 for the OTU table used in this study.

We used a linear model to correct for several patient and tumor covariates, individually as well as in combination, including patient age, sex, tumor stage, and tumor site. None of these factors, alone or in combination, were found to have a significant impact in this sample set. We also performed principle coordinate analysis using the difference between the tumor and normal abundances for each taxon. Using this unsupervised approach, there was no clear segregation of the patients by age, sex, tumor stage, site, or microsatellite instable (MSI) status. Additionally, we focused specifically on *Providencia* and *Fusobacterium*, and while there was a slight trend toward higher tumor stage with increases in these two genera at the tumor site, it was not statistically significant. We note that microsatellite instable/microsatellite stable (MSI/MSS) statuses were only available for 13 of the 44 patients.

Correlation analysis was performed using SparCC, available at [43] from Jonathan Friedman at MIT, on the complete OTU table collapsed to the genus level [44]. Pseudo *p* values were inferred using 100 randomized sets. Correlations with pseudo *p* values ≤0.05 that were within two degrees of separation from *Providencia* or *Fusobacterium* with absolute correlations of 0.05 or more were visualized using Cytoscape v.3.1.0 [45].

The PICRUSt v.1.0.0 pipeline was used to generate a virtual metagenome using the OTU table containing raw counts generated in the previous analyses by QIIME [29, 39, 40]. Pathways and enzymes were assigned using the Kyoto Encyclopedia of Genes and Genomes (KEGG) database options built into the pipeline. Virulence genes were identified by mapping the data in the PICRUSt enzyme abundance table to MVirDB using the UniProt database file, idmapping.dat, available from [46], as a key. See Additional files 3 and 4 for the metabolic enzyme and pathway abundance tables, respectively.

## Results

### Tumor microenvironments harbor microbiomes distinct from those of normal tissue microenvironments

**Fig. 1:**
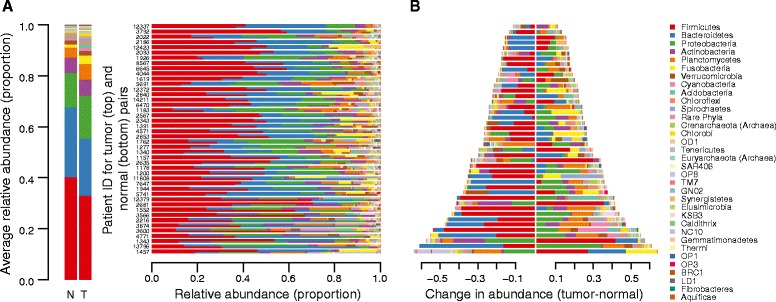
Differences in bacterial and archaeal phyla within the normal and colorectal cancer microbiomes. **a** Stacked bar plots indicating the proportional abundances of microbial phyla that are present at ≥1 % in at least one sample. The averages across all normal samples (*N*) and tumor samples (*T*) are presented at the left. Rows are arranged as patient-matched tumor (top) and normal (bottom) pairs. Patient ID numbers are appended to the right of the row pairs. **b** The data in panel **(a)** presented as the difference in abundance (Tumor – Normal) for each phylum, sorted in the same patient order, where a value of 0 would indicate no difference. Note that the legend at the right is common to panels **(a)** and **(b)**

We obtained patient-matched normal and tumor colon tissue samples from the University of Minnesota Biological Materials Procurement Network (BioNet) from 44 patients (see Additional file 1 for sample information). We assessed the microbiome associated with each sample by Illumina sequencing across the V5-V6 hypervariable regions of the 16S rRNA gene (see “Methods” for details). This analysis showed variation in the bacterial phyla abundance when comparing the matched normal and tumor tissues (Fig. 1a). This variability is consistent with previous reports and demonstrates that there is indeed a cancer-associated signature in the tumor microbiome [6, 10, 16, 18, 34, 47, 48]. At the level of the phyla, each sample was dominated by Firmicutes, Bacteroidetes, and Proteobacteria. There were clear and significant changes in these phyla between the normal and cancer states, with the tumors showing an enrichment of Proteobacteria and a depletion of Firmicutes and Bacteroidetes (Fig. 1b). Also consistent with previous reports, we saw an increase in the phylum Fusobacteria in the tumor-associated microbiome (two-sided Wilcoxon signed rank test q ≤ 0.1 after false discovery rate (FDR) correction for multiple tests) [7, 10, 12, 34].

**Fig. 2:**
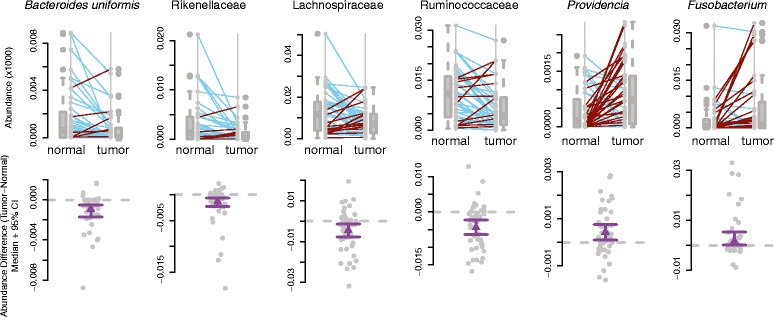
Differentially abundant taxa between matched normal and colorectal cancer microbiomes. Boxplots with corresponding paired dotplots indicating the relative abundances of several taxa showing differential abundance between tumor and normal samples. Lines connect the abundance in the normal (*left*) and tumor sample (*right*). Line colors indicate the directionality of the abundance change (*blue* and *red* for decreased and increased abundance in the tumor relative to the normal, respectively). Below we plot the difference between the tumor and normal abundance as *grey dots*, with the *purple line* representing the 95 % confidence (*95* % *CI*) interval and the mean. Values at 0 (*grey dotted lines*) represent no change between normal and tumor

**Fig. 3:**
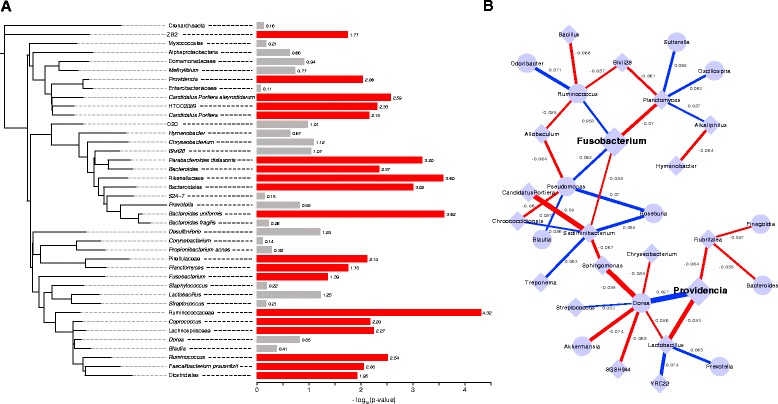
Relationships among the taxa found in colorectal cancer patients’ microbiomes. **a** Phylogenetic tree depicting the relatedness of the bacterial taxa present (>0.1 % of total) within 50 % or more of the samples. The bars to the right indicate the –log_10_(*p* value) from the Wilcoxon signed rank test to determine differential abundance between the normal and tumor microbiomes. *Red bars* indicate significance at 10 % FDR, while *gray bars* indicate that the specific taxon did not reach significance. **b** Correlation network showing the relationship among the abundances of genera with absolute values of 0.05 or more and statistical significance (pseudo *p* value <0.05). Edges indicate correlations: the edge thickness represents the magnitude and the color represents the sign (*blue* is positive correlation, *red* is negative correlation). Each node is a microbial genus where *diamond shaped nodes* indicate a higher average abundance and *circular nodes* indicate a lower average abundance in the tumor microbiome compared with normal

When we assessed the differences at the level of OTUs we discovered numerous changes between the normal and tumor microbiomes with significant differences in the abundances of 19 different taxa (Wilcoxon signed rank test q ≤ 0.1 after FDR correction; Additional file 5). Of note, the tumors showed decreases in the abundances of several taxa within the order Chlostridales, namely, Lachnospiraceae, Ruminococcaceae, and *Faecalibacterium prausnitzii*, as well as several members within the order Bacteroidales, including *Bacteroides*, Rikenellaceae, and *Bacteroides uniformis* (Figs. 2 and 3a). Taxa that were enriched in the tumor microbiomes included *Fusobacterium* and several Proteobacteria genera, including *Candidatus*, *Portiera* and *Providencia* (Figs. 2 and 3a; Additional file 6). Both *Fusobacterium* and *Providencia* are known pathogens, and when a correlation network is generated, it is clear that there are correlated abundance changes in the microbiome as a function of their presence (Fig. 3b). For instance, *Fusobacterium* species have been shown to have a mutualistic relationship with some *Pseudomonas* species at abscesses [49]. This co-occurrence is seen in our data as a positive correlation between the abundances of the two genera (Fig. 3b). Other specific interactions between different bacterial taxa remain speculative. In the case of *Lactobacillus* in the human microbiome, it has been demonstrated that there can be reciprocal interference between species in this genus and other bacterial species in the form of competition for epithelial cell adhesion. As both *Lactobacillus* and *Providencia* utilize cell adhesion in their colonization of the human body, this may explain the negative correlation between the two genera in our dataset (Fig. 3b). While there was not a significant correlation between the relative abundances of *Fusobacterium* and *Providencia* in this analysis, we assessed the overlap among patients who showed increased levels of these genera at the tumor sites. Taken individually, *Fusobacterium* and *Providencia* were more abundant in the tumor microenvironment of 23 out of 44 and 28 out of 44 patients, respectively. Nineteen out of 44 patients showed increases in both of the genera in their tumor microenvironments with respect to their normal matched tissue microbiomes.

### CRC-associated microbiome diversity

We calculated alpha-diversity using a variety of metrics within each of the samples using QIIME [39]. Alpha diversity metrics that account for phylogenetic relationships between the OTUs show that the tumor microbiomes exhibited higher alpha diversity than those of the normal, patient-matched microbiomes (*p* = 0.029 by two-sided Wilcoxon signed rank test). This is also true when using alternative measures of diversity such as the Shannon’s index or the Inverse Simpson’s (*p* = 0.020 and 0.024, respectively, by Wilcoxon signed rank test; Additional file 7).

### Variation in the functional pathways and enzymes in the tumor microbiome

**Fig. 4:**
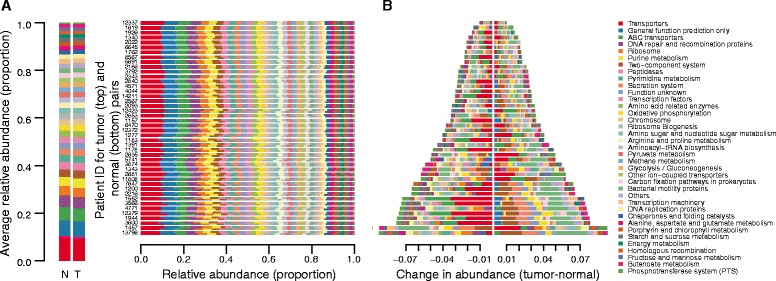
Differences in metabolic (KEGG) pathways within the normal and colorectal cancer microbiomes. **a** Stacked bar plots indicating the proportional abundances of metabolic pathways present at ≥1 % in at least one sample. The averages across all normal samples (*N*) and tumor samples (*T*) are presented at the left. Rows are arranged as patient-matched tumor (*top*) and normal (*bottom*) pairs. Patient ID numbers are appended to the right of the row pairs. **b** The data from panel (**a**), presented as the difference in abundance (Tumor – Normal) for each phylum, sorted in the same patient order, where a value of 0 would indicate no difference. Note that the legend at the right is common to panels (**a**) and (**b**)

A recent report presented the validation of a pipeline that leverages knowledge of the human gut reference genomes to predict general microbiome function and enzyme composition from 16S rRNA gene sequencing data [29]. While this approach is not suitable for making conclusive statements regarding single, specific enzymes, it is appropriate for general functional comparisons between groups of samples, as is the case in this report. Using this validated pipeline — Phylogenetic Investigation of Communities by Reconstruction of Unobserved States (PICRUSt) — we constructed a virtual metagenome for each of the samples’ microbiomes [29]. The KEGG database was used as a reference to determine the abundances of metabolic pathways and enzymes within the virtual metagenomes [50, 51]. As with the bacterial phyla, we saw significant variation in the predicted functional pathways represented within each of the sampled microbiomes (Fig. 4a), though, as expected from previous studies, we find that the variability in phylum abundances is far greater than the variability in the functional pathways (Fig. 4b) [2, 52]. It is important to note that the results of this analysis are predictions only and not direct measurements of sequences that correspond to pathway member or enzyme genes. Despite the validation of this prediction approach, it is possible that this method biases the predictions toward microbial genomes that are well documented to the exclusion of other, unknown or poorly documented taxa.

These observations highlight the substantial functional redundancy across the phyla. In other words, diversity among the taxa within a given microbiome can mask the functional similarities — taxonomically distinct microbes in the gut can operate identically, or nearly so, at the functional level. The patient-by-patient variability in phyla does not perfectly correspond to that seen at the functional pathway level, though, as expected, analysis at the level of enzymes and pathways provides insights that analyses of the taxa alone may not be able to identify, due to the high level of between-sample variation. In general, the differences seen at the pathway level are roughly an order of magnitude less than the differences seen at the phylum level (compare Figs. 1b and 4b). Although the pathway differences are smaller than those at the level of the phylum, there remain physiologically relevant, statistically significant changes between the normal and tumor metagenomes.

**Fig. 5:**
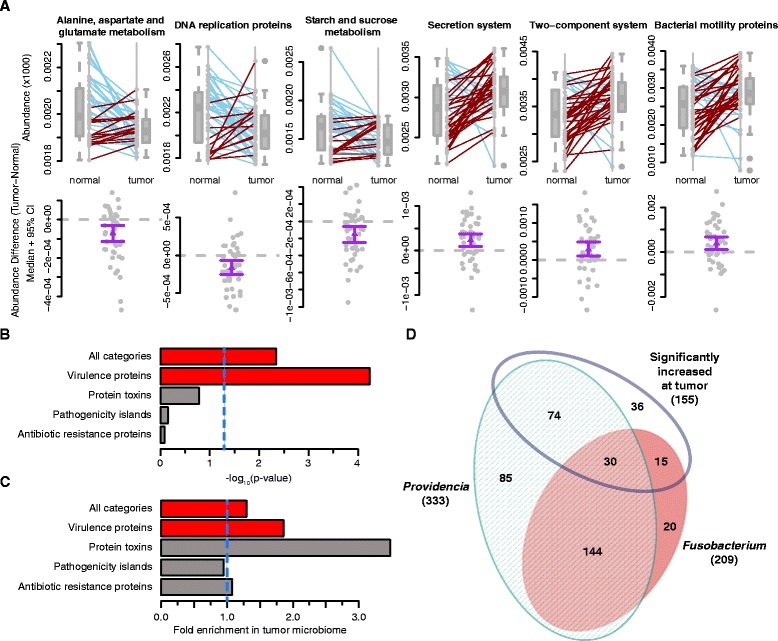
Differentially abundant pathways and enzyme classes between matched normal and colorectal tissue microbiomes. **a** Boxplots with corresponding paired dotplots indicating the relative abundances of several pathways showing differential abundance between tumor and normal samples. Lines connect the abundance in the normal (*left*) and tumor sample (*right*). Line colors indicate the directionality of the abundance change (*blue* and *red* for decreased and increased abundance in the tumor relative to the normal, respectively). Below we plot the difference between the tumor and normal abundance as *grey dots*, with the *purple line* representing the 95 % confidence interval (*95 % CI*) and the mean. Values at 0 (*grey dotted lines*) represent no change between normal and tumor. **b** Barchart showing the *p* values (−log_10_ transformed) obtained from Fisher’s exact test used to determine virulence category enrichment in the tumor-associated microbiome on the x-axis with the gene categories labeled on the y-axis. *Red bars* indicate significance by Fisher’s exact test (*p* < 0.005) and *gray bars* indicate no statistical significance. The *blue dashed line* indicates the standard significance cutoff of *p* = 0.05. **c** Barchart from the analysis in panel (**b**), demonstrating the fold-enrichment of virulence protein-encoding genes in the tumor-associated microbiome. The x-axis is the fold enrichment of the different virulence enzyme classes within the tumor microbiome relative to the normal microbiome. The *vertical blue dotted line* placed at 1 indicates the point where there is no difference between the normal and tumor microbiomes. **d** Venn diagram indicating the numbers of shared virulence-associated genes among *Providencia*, *Fusobacterium*, and the set of statistically significantly increased abundance genes at the tumor

Twenty pathways (as defined by KEGG, level 3) were found to be differentially abundant between the tumor and normal tissue. Alanine, aspartate, and glutamate metabolism, DNA replication proteins, and starch and sucrose metabolism were significantly depleted in the tumor microbiome (q ≤ 0.01 for each pathway by two-sided Wilcoxon signed rank test after FDR correction; Fig. 5a). Conversely, secretion system, two-component system, and bacterial motility protein pathways were significantly enriched in the tumor microbiome (q ≤ 0.04 for each pathway by two-sided Wilcoxon signed rank test after FDR correction; Fig. 5a).

To more closely examine the variation in the microbiome virulence potential, we used information regarding virulence association from MVirDB to annotate the predicted enzymes [53]. We found that the tumor-associated microbiome is significantly enriched with enzymes related to microbial virulence. We found this enrichment to be significant when including all possible virulence categories (*p* = 0.0046 by Fisher’s exact test; Fig. 5b, c). Additionally, when assessing enrichment for virulence-related genes by known functional categories in MVirDB, the tumors were significantly enriched for genes encoding general virulence proteins (*p* = 5.8 × 10^−5^, by Fisher’s exact test). Genes encoding bacterial toxins were also found at higher abundance in the tumor, but the enrichment was not statistically significant (*p* = 0.17 by Fisher’s exact test), likely due to low total gene counts for some categories (e.g., protein toxins).

We predicted that this virtual enrichment was driven by known pathogenic bacteria within the microbiome, i.e., *Providencia* and *Fusobacterium*. In fact, when a comparison is made among the virulence-associated genes significantly differentially found in the tumor microenvironment (155), the virulence genes associated with *Providencia* (333), and the virulence genes associated with *Fusobacterium* (209), there is substantial overlap among and between the three different groups (Fig. 5d). In order to exclude the possibility that the virulence enrichment and corresponding overlap between the virulence genes found at the tumor site were the result of non-specific effects rather than due to the potential contributions of *Providencia* and *Fusobacterium*, the entire PICRUSt pipeline and subsequent enrichment analyses were repeated using an OTU table from QIIME with both *Fusobacterium* and *Providencia* explicitly excluded. In this case, while there were still 123 virulence genes from other taxa associated with the tumor microbiome, the enrichment is not significant compared with the background of normal tissue from the same cancer patients (Fisher’s exact test one-sided *p* value >0.9). This demonstrates that the virulence signature in the microenvironment is dependent on *Fusobacterium* and *Providencia*.

To highlight the differences seen when assessing patient-matched tissue samples compared with assessing case and control stool samples, we performed a comparison of our results with those of Zackular et al. [11]. Zackular et al. performed an assessment of the microbiomes of CRC patients’ stool samples relative to those of normal patients or patients with colorectal adenomas [11]. In comparison with the data presented here, a prediction would be that both *Fusobacterium* and *Providencia* would be found at increased abundances in CRC patients relative to normal controls. While the data from Zackular et al. show a statistically significant increase in *Fusobacterium* in CRC patients' stools, *Providencia* at the genus level was not detected in the stool samples. However, there was a clear trend showing a doubling of the abundance of Enterobacteriaceae (the family to which *Providencia* belongs) when looking at stools from normal patients in comparison with stools from patients with adenomas and yet again when comparing adenoma patients and CRC patients. A likely explanation for the difference between our study and Zackular et al. is that data in the latter study were collected from stools and were not patient-matched. Thus, inter-individual variability likely decreased the ability to identify some tumor-associated taxa, such as *Providencia*.

## Discussion

At the phylum level, the differences seen between the normal and tumor tissue-associated microbiomes are consistent with many previous reports [6, 10, 16, 18, 34, 47, 48]. When assessing the data using information that accounts for more fine-grained detail with respect to taxonomy, we have made several important findings. Two of the genera we found to be enriched in the tumor microbiome, *Providencia* and *Fusobacteria*, are known to be pathogenic; *Fusobacteria* has been implicated previously in CRC [7, 10, 12, 34].

Species belonging to the genus *Providencia* have been implicated as infectious agents causing urinary tract infections, ocular infections, and gastroenteritis [37, 54–60]. In addition, it is a genus in which several sub-strains have acquired resistance to commonly used antibiotics [56, 61, 62]. *Fusobacterium* is a genus that encompasses several species known to be pathogenic in humans; they are obligate anaerobes, with known sites of infection in the oral cavity as well as in the gastrointestinal tract [63, 64]. The finding that these particular genera are prevalent in the tumor microenvironment suggests several alternative, though not mutually exclusive, hypotheses. One possibility is that these bacteria are causative in oncogenesis or tumor progression; another possibility is that these species are being enriched as the tumor has formed a niche that favors these bacteria. In the case of *Fusobacteria*, the results from several different studies, both correlative and mechanistic, indicate that it is likely a cancer driver [7, 14, 34]. In the case of *Providencia*, there are as yet no definitive studies that implicate this genus as a contributor to CRC. The discovery of *Providencia* in the tumor microbiome is interesting as, similar to *Fusobacteria*, it encodes a potent, immunogenic lipopolysaccharide [58, 65]. In fact, several virulence genes responsible for lipopolysaccharide biosynthesis are shared by both genera and are also significantly increased in the tumor microenvironment (Fig. 5d). A recent study, using *Drosophila* as a model system, performed a genomic comparison of four different species of *Providencia* isolated from the human gut [66]. These researchers demonstrated that these four species share common sets of virulence-related genes, including a type 3 secretion system and genes for cell adhesion. Additionally, *Providencia* has been shown to disrupt the epithelial membrane in the intestines, though the mechanism by which this is accomplished is still unclear [55, 58, 67, 68]. These factors manifest phenotypically as gastroenteritis, though with our discovery of its association with the cancer microenvironment, it is a promising candidate cancer-promoting pathogen [60].

From a diagnostic and therapeutic perspective, assessing the CRC-associated microbiome by testing for differentially abundant taxa is an eminently worthwhile endeavor as it is the logical location to look for specific taxa that could be biomarkers and/or targets for intervention in CRC. However, it is possible that the search for specific taxa might miss the larger perspective. For instance, as described above, *Fusobacteria* and *Providencia* share many important phenotypic characteristics — potent, immunogenic lipopolysaccharide and the ability to damage colorectal tissue. These similarities might be better assessed using metagenomic or metatranscriptomic approaches, virtual or otherwise, as these key features are undoubtedly reflected in the genes that these particular bacteria encode, many of which are shared virulence factors with known detrimental properties.

Defining a clear set of virulence factors a priori as targets of interest and assessing their relative expression is a promising approach to CRC therapy. This report proposes such an approach by showing the striking predicted enrichment of virulence genes in the tumor-associated microbiome, potentially driven by *Fusobacteria* and *Providencia*. The fact that virulence proteins are predicted to be enriched in the tumor-associated microbiome lends support to the hypothesis that the microbiome is an active contributor to CRC and not just a passive byproduct of the changes the tumor makes in the organ. In the case of *F. nucleatum*, it is clear that there is a direct functional link between the bacteria and cancer development, though more work using cell culture and model organisms will be needed in the future to empirically assess the mechanistic interplay between colorectal tissues and specific components of the microbiome [14]. It is important to note that this clear enrichment is likely underestimated because MVirDB, while expansive, does not currently encompass all known virulence genes in the microbiome, and, as the field of medical microbial genomics advances, new virulence genes will undoubtedly be discovered. For instance, the FadA protein from *F. nucleatum* has been reported as a critical virulence factor, yet as it is a recent report, this finding has not yet made its way into MVirDB as of this submission [14, 53].

It is important to note that this research uses 16S rRNA gene sequences as the starting point. Although this approach has obvious benefits in terms of resource expenditures and computational processing, there are several potential disadvantages. First, the microbiome functional assessment presented here uses a prediction method that, while validated and robust when applied to human gut samples, remains a prediction and may not necessarily perfectly reflect the biological reality [29]. Another concern, as with all DNA-based approaches, is that even when a gene is predicted in a sample, it may still not be expressed or active. Additional metatranscriptomic research will undoubtedly shed light on this situation in the future. We also note that although our results show microbiome patterns with potential roles in cancer, we cannot determine causality as part of this study. We expect follow-up studies to focus on assessing the causal role of the microbiome in colon cancer using animal models and cell culture systems.

## Conclusions

It is clear that there are numerous taxa in the CRC microbiome that are correlated with the disease. Here, in addition to the previously reported genus *Fusobacterium*, we report the discovery of another genus with similar pathogenic features, *Providencia*. This manuscript also presents an analysis that incorporates predicted information at the functional (e.g., virulence potential) level to assess differences between the normal and cancer-associated microbiomes. It is important to note that these two approaches (taxonomy-based and function-based) provide different, yet interdependent information about the microbes in the tissue microenvironment. Our work demonstrates that utilizing this combined approach can provide researchers with specific taxa as biomarkers and/or therapeutic targets while also looking globally at the predicted pathogenic potential of the microbiome and showing a clear predicted enrichment of virulence-associated microbial genes present in the CRC microbiome. As with the bacterial genera associated with the disease, these virulence genes may provide researchers and clinicians with targets for therapeutic intervention to improve patient outcomes.

## Additional files

Additional file 1:
**Sample and patient clinical information and metadata.** A tab-delimited file containing a sample map containing patient and tumor clinical information as well as the specific sample numbers that will allow proper identification of tumor and normal matched pairs. Additional file 2:
**Unfiltered OTU table.** A tab-delimited file containing the unfiltered OTU table with proportional abundances of the taxa output from the QIIME pipeline. Additional file 3:
**Enzyme abundances.** A tab-delimited file containing the unfiltered proportional abundances of enzymes detected in the virtual metagenomes of each sample using PICRUSt. The file also contains an additional column with an annotation from MVirDB, when available, as well as a column that indicates if the predicted enzyme was significantly more abundant at the site of the tumor. Additional file 4:
**Pathway abundances.** A tab-delimited file containing the unfiltered level 3 KEGG pathway proportional abundances for each sample generated using PICRUSt. Additional file 5:
**Taxon significance testing.** A tab-delimited file containing *p* values, q values, and the site at which the taxa are more abundant for the taxa tested for significance after correcting for multiple tests. Those included in this analysis were those taxa that were present in at least 50 % of all samples at an abundance of >0.1 %. Additional file 6:
**Genus-specific PCR and** ***FadA*** **qPCR.** **a** Genus-specific PCR for *Fusobacterium*. Genus-specific PCR was carried out for a subset of samples for normal and tumor-matched DNA samples. A PCR reaction containing only water rather than DNA was used as a negative control, while DNA from the ATCC control strain *Fusobacterium nucleatum* 25586 was used as a positive control. Bands on the 2 % agarose TAE gel are visible for both the positive control as well as for the tumor samples at the expected amplicon size of 162 base pairs. **b** Genus-specific PCR for *Providencia*. Genus-specific PCR was carried out for a subset of samples for normal and tumor matched DNA samples. A PCR reaction containing only water rather than DNA was used as a negative control, while DNA from the ATCC control strain *Providencia alcalifaciens* 9886 was used as a positive control. Bands on the 2 % agarose TAE gel are present for both the positive control as well as for the tumor samples at the expected amplicon size of 515 base pairs. **c** *FadA* qPCR. A subset of samples was used to determine the relative abundance of the *FadA* gene in tumor samples relative to normal samples. Both the normal and tumor samples were normalized internally to the total abundance of eubacteria. The value for the normalized tumor *FadA* abundance was divided by the normalized value for normalized normal *FadA* abundance to arrive at the fold differences indicated. Additional file 7:
**Microbial diversity within normal and tumor-associated microbiomes.** Paired line plots show the phylogenetic diversity, Shannon’s Index, and Inverse Simpson’s Index (alpha diversity metrics) for the microbiomes associated with normal and patient-matched tumor samples. The colors of the lines represent the direction of the change for each matched pair (*blue lines* indicate a decrease in diversity from normal to tumor, while *red lines* indicate an increase in diversity from the normal to tumor). *P* values were calculated using a two-sided Wilcoxon signed rank test.

## Abbreviations

CRC: colorectal cancer
FDR: false discovery rate
KEGG: Kyoto Encyclopedia of Genes and Genomes
OTU: operational taxonomic unit
PCR: polymerase chain reaction
PICRUSt: Phylogenetic Investigation of Communities by Reconstruction of Unobserved States
qPCR: quantitative PCR
SCFA: short chain fatty acid
