# Supplementary figures and images for: Virulence genes are a signature of the microbiome in the colorectal tumor microenvironment
Source: Genome Med. 2015 Jun 24;7(1):55. doi: 10.1186/s13073-015-0177-8 (PMC4499914; doi:10.1186/s13073-015-0177-8)

**A***Fusobacterium* genus-specific PCR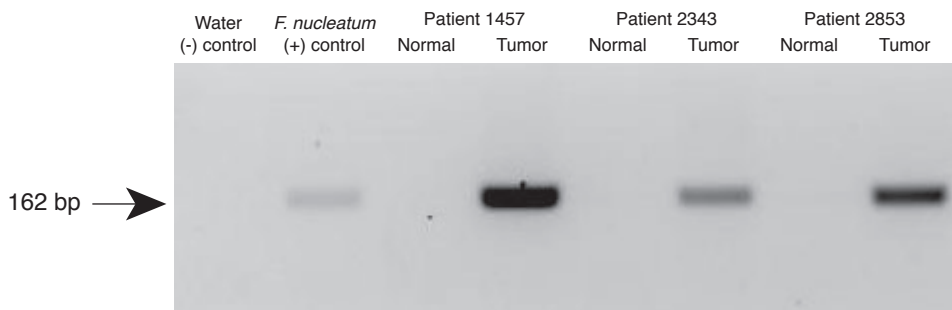**B***Providencia* genus-specific PCR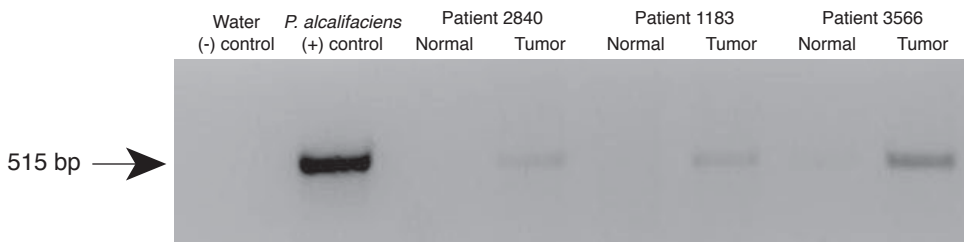**C**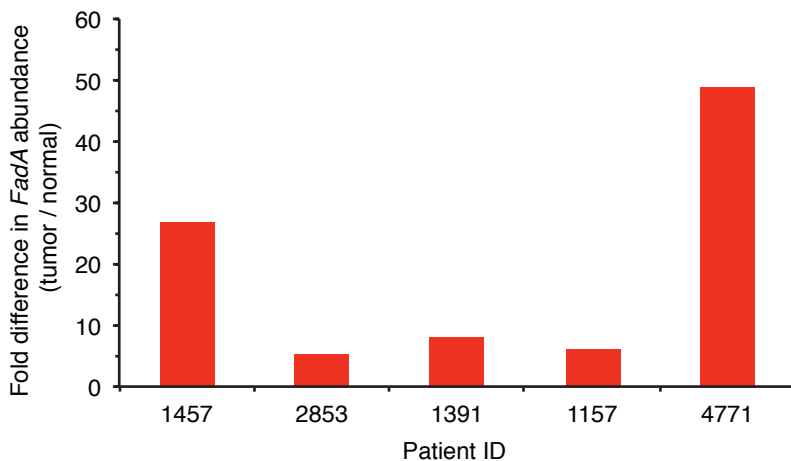

Supplement: Additional file 6: — Genus-specific PCR and FadA qPCR. a Genus-specific PCR for Fusobacterium. Genus-specific PCR was carried out for a subset of samples for normal and tumor-matched DNA samples. A PCR reaction containing only water rather than DNA was used as a negative control, while DNA from the ATCC control strain Fusobacterium nucleatum 25586 was used as a positive control. Bands on the 2 % agarose TAE gel are visible for both the positive control as well as for the tumor samples at the expected amplicon size of 162 base pairs. b Genus-specific PCR for Providencia. Genus-specific PCR was carried out for a subset of samples for normal and tumor matched DNA samples. A PCR reaction containing only water rather than DNA was used as a negative control, while DNA from the ATCC control strain Providencia alcalifaciens 9886 was used as a positive control. Bands on the 2 % agarose TAE gel are present for both the positive control as well as for the tumor samples at the expected amplicon size of 515 base pairs. c FadA qPCR. A subset of samples was used to determine the relative abundance of the FadA gene in tumor samples relative to normal samples. Both the normal and tumor samples were normalized internally to the total abundance of eubacteria. The value for the normalized tumor FadA abundance was divided by the normalized value for normalized normal FadA abundance to arrive at the fold differences indicated. [file 13073_2015_177_MOESM6_ESM.pdf]

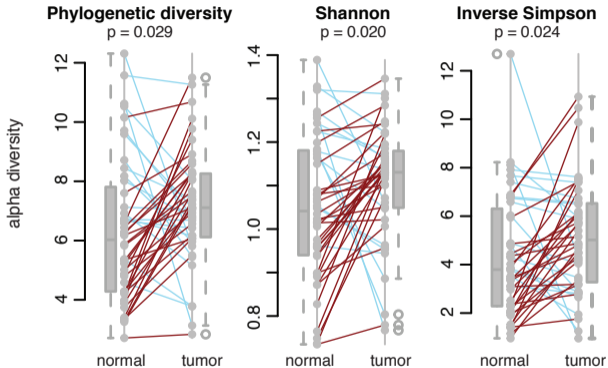

Supplement: Additional file 7: — Microbial diversity within normal and tumor-associated microbiomes. Paired line plots show the phylogenetic diversity, Shannon’s Index, and Inverse Simpson’s Index (alpha diversity metrics) for the microbiomes associated with normal and patient-matched tumor samples. The colors of the lines represent the direction of the change for each matched pair (blue lines indicate a decrease in diversity from normal to tumor, while red lines indicate an increase in diversity from the normal to tumor). P values were calculated using a two-sided Wilcoxon signed rank test. [file 13073_2015_177_MOESM7_ESM.pdf]
